# The potential of methylation signal detection in eDNA toward functional ecological monitoring

**DOI:** 10.1038/s42003-026-10496-2

**Published:** 2026-06-20

**Authors:** Chengbin Liu, Xia Fei, Itsuki T. Hirayama, Genfu Yagi, Masayuki Ushio

**Affiliations:** 1 Department of Ocean Science, The Hong Kong University of Science and Technology, Clear Water Bay, Kowloon, Hong Kong SAR China; 2 Department of Integrated Biosciences, Graduate School of Frontier Sciences, The University of Tokyo, Kashiwa, Chiba, Japan; 3 Institute of Life and Environmental Sciences, University of Tsukuba, Tennodai, Tsukuba, Ibaraki, Japan; 4 Faculty of Science, University of the Ryukyus, Senbaru, Nishihara, Okinawa, Japan

**Keywords:** Conservation biology, Ecosystem ecology, Molecular ecology

## Abstract

Environmental DNA technology has revolutionized biomonitoring, primarily capturing the presence/absence of target taxa. Recent advances have revealed that eDNA also retains epigenetic signatures. In this perspective, we focus on DNA methylation signals in eDNA, which we define as meth-eDNA, and analyze three pivotal topics: (1) Detection of methylation signals in eDNA, (2) stability in aquatic environment, and (3) applications as ecological indicators. This perspective highlights the potential of meth-eDNA in non-invasive population-level trait inference. Future integration with multi-omics and sequencing innovations will achieve more precision in ecosystem conservation and management.

## Abstract (web summary)

A Perspective introduces meth-eDNA, using DNA methylation in environmental DNA to infer population traits. Emerging methods could enable noninvasive inference of age, sex ratio, reproduction and stress, expanding eDNA from species detection to functional ecosystem monitoring.

## Introduction

Environmental DNA (eDNA) refers to genetic material obtained directly from environmental samples, such as water, soil, and air^1–3^. Organisms continuously release eDNA into the environment, which accumulates in various forms^4^. eDNA may include free-floating DNA or intracellular, microbial DNA, or may originate from cellular debris shed, such as hair, feces and pollen^5–7^. Because eDNA may contain information about organisms inhabiting the environment, it has been utilized for monitoring biodiversity for nearly 20 years^8^. While the term eDNA may indicate a wide range of physical forms of DNA in the environment, we define eDNA as DNA derived from macrobial organisms such as fish and tetrapods in this perspective and exclude microbial DNA to clarify the scope. Also, our definition includes DNA obtained not only from environmental substrates such as water and soil but also from other biological materials found in the environment, including feces and shed tissues. We focus primarily on aquatic eDNA because most empirical meth-eDNA studies to date have been conducted in aquatic systems. While airborne and soil-derived eDNA show promise, research in these areas remains limited. Furthermore, interpreting their methylation signals may be harder because of highly heterogeneous sources of DNA (particularly, soil eDNA) and the stronger effects of environmental parameters on preservation, degradation and source attribution^9,10^.

Biodiversity monitoring with eDNA-based methods typically start by collecting environmental samples, followed by molecular analysis. The downstream workflow diverges depending on the method used. Currently, quantitative PCR (qPCR) and eDNA metabarcoding are commonly utilized for eDNA analysis, whereas shotgun metagenomics is emerging. The method of qPCR amplifies species-specific genetic markers, enabling the detection and quantification of known target species eDNA^11–13^, while eDNA metabarcoding involves the amplification of informative marker genes, high-throughput sequencing, and taxonomic classification^14–17^. In contrast, shotgun sequencing randomly sequences all DNA fragments, mitigating PCR biases and revealing an unbiased view of community composition^18–22^. One of the major advantages of eDNA in biomonitoring is the ease of sample collection and its non-invasive nature. In aquatic monitoring, sampling, filtration, and preservation can be completed rapidly. Compared with traditional monitoring techniques such as seining^23^, electrofishing^24^, and visual observation (e.g., for marine mammals^25^), eDNA sampling is more time-efficient^26,27^. In addition, water samples can be collected without environmental disturbance^28^. These advantages enable more frequent and spatially extensive sampling, improving the ability to detect invasive species^29^ and assess temporal changes in community composition^30–32^.

Nonetheless, the information that eDNA can provide is still limited; for example, eDNA studies are often focused on presence/absence detection^33,34^ and species identification^35,36^. Current eDNA methods do not allow access to individual-level traits, including age, sex, or physiological condition^37^. Assessments of reproductive based on eDNA patterns often rely on detection of a sharp increase in eDNA concentration and/or changes in the ratio of mitochondrial and nuclear DNA, which is not direct evidence for the release of reproductive cells (e.g., sperm)^38,39^.

Recently, new eDNA techniques have been developed to detect more information about biodiversity, for example, intra-specific diversity^9^, community composition^19,40^ and biomass estimation^41–43^. Among these new directions, the detection and interpretation of DNA methylation pattern in eDNA represent a promising direction in detecting more functional signatures in eDNA research.

### Epigenetics and DNA methylation

Epigenetics is the study of heritable changes in gene expression that occur without alterations to nucleotide sequence^44^, including transcription factors, noncoding RNAs, DNA methylation, and histone modifications^45^. We specifically focus on DNA methylation in this perspective paper, as it is the most accessible epigenetic signal in the context of eDNA.

DNA methylation predominately refers to the covalent addition of a methyl group (–CH_3_) to the 5th carbon of cytosine in DNA, forming 5-methylcytosine (5mC), primarily at cytosine-phosphate-guanine dinucleotides (CpG; p indicates a phosphodiester bond)^46,47^. In addition to cytosine methylation, methyl group can also occur on adenosine (N^6^-methyladenine, 6 mA), although its functional significance in many eukaryotes remain debated^48^. DNA methylation is regulated by DNA methyltransferase enzymes (DNMTs). DNMT1 is responsible for maintaining existing methylation patterns during replication, while DNMT3A and DNMT3B establish de novo methylation at new loci^49^. Moreover, methylation is reversible and is mediated by ten-eleven translocation (TET) family enzymes (TET1, TET2, and TET3), which oxidize 5mC to 5-hydroxymethylcytosine (5hmC), and subsequently to unmethylated cytosine through base excision repair^50^. Genomic regions with a high frequency of CpG dinucleotides are called CpG islands. They are frequently located in or near gene promoter regions^51^. DNA methylation plays key roles in transcriptional silencing, mostly through promoter hypermethylation, X-chromosome inactivation and genomic imprinting (a mechanism that enforces monoallelic gene expression)^47,52^.

Previous research has shown methylation landscapes exhibit high cell-type specificity and tissue dependence^53,54^. DNA methylation plays a pivotal role in the phenotypic prediction of various human diseases^55–57^. Importantly, it also serves as a powerful signal of age, reflecting both biological processes and accumulation of changes over time. Specific CpG sites undergo highly predictable, often linear, methylation changes that are tightly correlated with chronological age^58,59^. Moreover, DNA methylation patterns show sex-specific differences that are detectable across tissues and species. Numerous CpG sites, particularly on the X chromosome but also on autosomes, display consistent sex-associated methylation patterns from birth^60,61^, although X-chromosome inactivation (Lyonization) is restricted to mammals and some rare exceptions (e.g., particular aphid species). These epigenetic signatures are implicated in sex-biased gene expression and contribute to developmental, metabolic, and disease-related dimorphisms^62,63^. The role of DNA methylation in the reproductive process is primarily reflected in regulating gene expression during gonad development^64^, responding to environmental changes^65,66^, and thus regulating germ cell formation^67^. Especially before and after spawning, dynamic changes in methylation status determine whether reproductive function is activated or suppressed^68,69^. This mechanism is not only crucial for individual physiological regulation but also in ecological species protection.

**Fig. 1:**
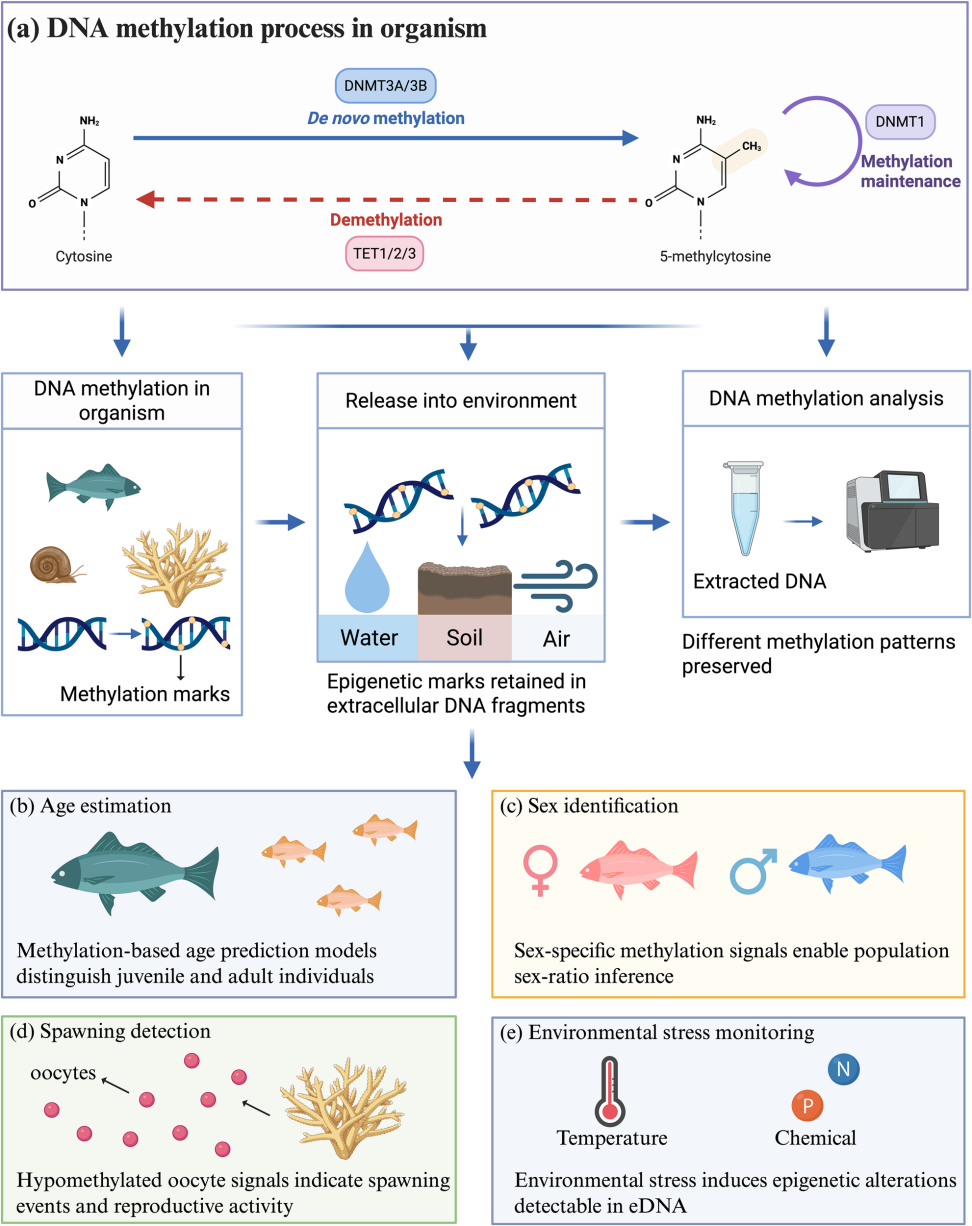
The conceptual explanation of DNA methylation and potentials of meth-eDNA to detect detailed characteristics of target organisms. **a** A simplified mechanism of DNA methylation, showing the conversion of cytosine to 5 methylcytosine and reversible demethylation process. Using cytosine as an example, DNA methylation is an epigenetic modification in which cytosine is converted to 5-methylcytosine by DNA methyltransferases and can be passively diluted during replication or actively removed through TET-mediated oxidation and subsequent base excision repair. Methylated nucleotides can be retained and detected in eDNA. Based on these mechanisms, meth-eDNA enables four potential ecological applications: **b** Age structure assessment through methylation-based epigenetic clocks; **c** Sex ratio estimation via sex-specific methylation signals; **d** Spawning detection by identifying germ cell-derived demethylation signatures; and **e** Stress and health monitoring. These advances transform eDNA from a taxonomic census tool into a functional phenotyping platform, revealing mechanisms underlying biodiversity dynamics such as reproductive timing, demographic shifts, and stress responses. Figure 1 was created in BioRender. Chengbin, L. (2026) https://BioRender.com/cgjh7xu.

Detecting methylation signals in eDNA has the potential to overcome several limitations of conventional eDNA-based biodiversity monitoring (shown as Fig. 1)^37^. Here, we define meth-eDNA as DNA methylation signals in eDNA fragments under our definition of eDNA. Meth-eDNA provides additional biological information beyond the nucleotide sequence typically analyzed in conventional eDNA studies. In this perspective, we will introduce the following: (1) how to measure DNA methylation rates, (2) existing examples of the detection of meth-eDNA, and (3) potential applications of meth-eDNA. Based on these, we will discuss future direction of meth-eDNA.

## Methods for detecting DNA methylation

The development of DNA methylation detection technologies is closely tied to both molecular detection platforms and computational modeling strategies. Most studies are on CpG islands due to methodological limitations, although recent advances allow methylation analyses beyond. Currently, most techniques that detect DNA methylation include methylation-specific polymerase chain reaction (MSP), whole-genome bisulfite sequencing (WGBS), reduced representation bisulfite sequencing (RRBS), targeted bisulfite sequencing including bisulfite amplicon sequencing (BSAS) and pyrosequencing, site-specific techniques including methylation-sensitive high-resolution melting (MS-HRM) and 5-Methylcytosine binding domain (MBD) sequencing^70–76^. In addition, third-generation sequencing technologies, such as Pacific Biosciences (PacBio) single-molecule real-time sequencing and Nanopore sequencing, are increasingly being employed for DNA methylation analysis^77,78^. These methods can directly detect methylated nucleotides without bisulfite conversion. Basically, methods for DNA methylation can be categorized along two axes: chemistry (bisulfite vs. non-bisulfite) and scope (genome-wide vs. locus-specific). We will introduce them in the following sections and Table 1.

**Table 1:** Pros and cons of different methods for studying DNA methylation

Methods (Bisulfite)	Resolution Scope and Context	Pros	Cons	Reference
Whole-Genome Bisulfite Sequencing (WGBS)	Genome-wide -5mC	• Single-base resolution • Ideal for comprehensive methylation mapping	• Expensive • Requires high input DNA • Computationally intensive	^76,134^
Reduced Representation Bisulfite Sequencing (RRBS)	Genome-wide -5mC	• Cost-effective • Suitable for transcriptional regulation studies	• Limited genome coverage • Bias towards certain regions	^82,135^
Methylation-specific polymerase chain reaction (MSP)	Loci-specific -5mC	• High sensitivity • Low‑put DNA • Technically easy	• Depends on primer design • Susceptible to PCR contamination • Hard to detect minor methylation differences	^70,136^
Bisulfite Amplicon Sequencing (BSAS)	Loci-specific -5mC	• High sensitivity • Scalable for multiple loci • Efficient for targeted analysis	• Requires sequencing infrastructure • Limited to predefined regions	^71,137^
Methylation-Sensitive High-Resolution Melting (MS-HRM)	Loci-specific -5mC	• High sensitivity (detects ~0.1% methylation) • Low cost • PCR-based, simple setup Suitable for rare event detection	• Semi-quantitative Requires careful primer design • Only leads an average methylation level across the amplified region.	^73,138^

Methods (non-bisulfite)	Resolution Scope and Context	Pros	Cons	Reference
5-methylcytosine binding domain (MBD)	Genome-wide -5mC	• Low-cost dsDNA enrichment • Reliable (high cross-assay concordance)	• CpG-density bias (preferentially captures CpG-rich methylated regions)	^75,139^
Nanopore-based methylation profiling	Genome-wide & Loci-specific -5mC, 5hmC and 6 mA	Detects methylation directly • No need for bisulfite conversion or amplification	• Requires specialized workflows to run methylation calling pipeline • Needs sufficient DNA without amplification • Expensive	^78,136,140^
PacBio-based methylation profiling	Genome-wide & Loci-specific -6ma, 4mc and 5mc	• No bisulfite conversion or amplification needed • Enables complete genome assembly	• High raw error rate (requires polishing) • Expensive and requires high DNA input • Costs for large-scale studies	^77,141^
Enzymatic Methyl-seq (EM-seq)	Genome-wide -5mc and 5hmc	• Non-destructive to DNA • High CpG detection • Effective with low-input and degraded samples-	• Does not detect 5fC or 5caC • Dependent on precise enzyme activity and reaction conditions • Slightly reduces detection in highly methylated DNA	^97,142^

Table 1 is organized based on two criteria (non-bisulfite-based vs. bisulfite-based, genome-wide vs. locus-specific) to elucidate the methodological landscape for eDNA methylation detection.

### Bisulfite-based methods

Bisulfite-based methods are one of the most widely used, which include reactions of unmethylated cytosine (C) converted to uracil (U) by sodium bisulfite. Methylated cytosines, however, are protected from this conversion and remain unchanged. Following bisulfite treatment, the DNA methylation patterns can be analyzed using sequencing-based methods or PCR-based methods. Using sequencing-based methods, researchers can identify which cytosines are methylated by aligning the bisulfite-converted sequence data with reference genome^79^. Among genome-wide bisulfite methods, WGBS offers single-base resolution across the entire genome^76,80^. Despite its resolution, WGBS is often cost-prohibitive for huge cohorts due to high per-sample sequencing depth and downstream compute needs. RRBS is more cost-effective and focused on CpG-rich regions, making it suitable for studies on transcriptional regulation^81,82^. However, it offers broad but sparse genomic representation, with limits CpG enrichment and low reproducibility across biological replicates.

Bisulfite-based methods also include several locus-specific assays, notably MSP, BSAS, pyrosequencing, and MS-HRM. MSP uses primers specific for bisulfite-modified methylated or unmethylated sequences. This allows for determination of the methylation status at target CpG sites or regions^70^. Its advantages include high sensitivity due to PCR-based discrimination and simple workflow adaptable to low-input or degraded samples, because nested MSP employs two successive amplifications to enrich scarce bisulfite-converted templates and improve target specificity, while multiplex MSP allows simultaneous amplification of multiple short amplicons. But it depends on proper primer design and complete bisulfite conversion, and is susceptible to PCR contamination and has limited ability to detect subtle methylation differences^70^. BSAS integrates next-generation sequencing with library preparation that contains locus-specific PCR enrichment after bisulfite conversion^71^. It has high sensitivity and cost-efficiency for multi-gene parallel DNA methylation analysis. Still, it is limited by poor coverage of large CpG islands and potential bias from heterogeneous tissue sources^83^.

Bisulfite-based pyrosequencing is also commonly used for many mammals^84–86^. Pyrosequencing is a method that detects real-time light signals from the release of pyrophosphate during nucleotide incorporation. Pyrosequencing accurately quantifies DNA methylation at specific CpG sites, making it a gold standard for regional methylation analysis^87^.

Another widely utilized technique is PCR based methods, such as MS-HRM, which has high sensitivity for detecting DNA methylation at specific loci^73^. Rather than directly sequencing bisulfite-converted DNA, MS-HRM employs high-resolution melting analysis to detect methylation-induced sequence alterations. This method is particularly suitable for screening applications and low-frequency methylation detection with minimal cost^88^. Its primer design, which ensures equal amplification efficiency for both methylated and unmethylated templates, can minimize PCR bias and allow the detection of rare methylation events at frequencies as low as 0.1%^73^. The limitation of MS-HRM is that methylation levels are not provided per CpG site, but only as an average across the amplified region.

### Non-bisulfite methods

Among non-bisulfite methods, MBD-based methods are widely used for genome-wide scale studies. Due to its methylation-specific DNA binding capacity, MBD can bind methylated DNA in vitro^75^. It supports DNA methylation profiling by using methyl-CpG-binding proteins to enrich methylated fragments, followed by next-generation sequencing and bioinformatic workflows. MBD-seq is a cost-effective method for obtaining genome-wide CpG methylation information, particularly suitable for large-scale research in fish breeding and nutrition^89^.

Third-generation sequencing technologies allow direct sequencing of single molecules^90^. It does not require chemical treatment or bisulfite conversion and enables base-resolution and quantitative methylation detection^77^. Among them, nanopore sequencing is a long-read DNA sequencing technology developed by Oxford Nanopore Technologies (ONT) that infers nucleotide identity and base modifications from characteristic changes in ionic current when DNA molecules pass through the nanopore. A major advantage of this approach is its ability to directly detect DNA methylation in all sequence contexts, including 5mC, 6 mA, and 5hmC, without chemical conversion or amplification^78^. Depending on the library preparation and enrichment, it supports both genome‑wide methylation profiling and locus‑specific analysis^91,92^. A nanopore sequencing-based protocol was developed to detect base modifications in eDNA molecules derived from water samples^92^. In addition, nanopore sequencing enables adaptive sampling for DNA fragments of at least 900 bp^93^. It combines real-time base calling and mapping to selectively identify and discard off-target reads during sequencing^94^.

PacBio-based methylation profiling is another method for detecting DNA methylation using Pacific Biosciences Single Molecule Real-Time (SMRT) sequencing. It identifies methylated bases by analyzing real-time kinetic signatures that are generated during DNA synthesis^95^. The approach can be applied genome‑wide using whole‑genome sequencing^77^, or locus‑specifically using PCR‑free CRISPR–Cas9^96^, enabling both comprehensive surveys and focused interrogation of selected regions.

A recent technology, named enzymatic methyl-seq (EM-seq), is a bisulfite-free, enzyme-based method for detecting 5mC and 5hmC at single-base resolution^97^. EM-seq offers high sensitivity, even coverage, and DNA integrity with low input requirements; however, it cannot detect 5fC/5caC and involves complex enzymatic steps. In addition, PCR‑free CRISPR–Cas9 enrichment is also a major approach in meta-epigenomic analyses particularly for microbial communities^96^. Both two methods can be coupled with both PacBio and Oxford Nanopore sequencing.

### Detection of DNA methylation signals in aquatic eDNA

Freshly shed eDNA likely retains methylation signals of the source organism, though these methylated cytosines are prone to deamination as the DNA degrades^98^. This hypothesis laid a theoretical foundation for detecting epigenetic signals in environmental samples. Following this, the years 2022–2023 saw the emergence of the first empirical studies that detected DNA methylation signals in aquatic systems (mainly in aquarium tanks). Zhao et al. conducted aquarium tank experiments using a freshwater snail (Lymnaea stagnalis), comparing methylation profiles between tissue-derived DNA and eDNA^99^. Tissue DNA was extracted from four life stages of snails, while eDNA was extracted from tank water, both of which were sequenced to assess methylation levels. Water eDNA exhibited clear and quantifiable DNA methylation patterns, which varied significantly across different developmental stages. This study provided the first experimental evidence that water eDNA carries information on population-level traits such as age structure. However, it remains unclear whether these methylation signals are stably retained in environmental samples. Recent studies have provided further evidence. Hirayama et al. conducted a tank experiment focused on the zebrafish species (Danio reri*o*) and demonstrated that even after partial degradation of eDNA in the environment, the methylation levels of eDNA closely mirrored those of the original somatic tissue DNA^100^. Additionally, they found no significant differences in methylation rates between tissue-derived DNA and eDNA. The study also investigated the effects of different cellular sources on the methylation signals. During the peak spawning period of fish, the water eDNA showed a high abundance of unmethylated DNA fragments, which could originate from germ cells. By focusing on specific DMRs, ecologically relevant information can be obtained from eDNA.

### Potential applications of meth-eDNA to ecological studies

Based on the pioneering studies that detected DNA methylation signals in environmental samples, researchers have begun exploring eDNA methylation to infer functional ecological information. Meth-eDNA, like eDNA, provides information at the population level rather than individual level because eDNA typically represents a mixture of DNA from multiple individuals. As a result, the detected epigenetic signals demonstrate an aggregate of biological traits across the population such as age, sex, reproductive state, and health status, making meth-eDNA is a promising tool for monitoring population-level functional attributes. Here, we introduce several potential applications of meth-eDNA to study ecological communities: (1) age structure inference; (2) sex identification with sex ratio monitoring; (3) reproductive state and spawning detection; (4) health status and stress responses.

### Age structure

DNA methylation changes predictably with chronological age, enabling the development of epigenetic clocks^101^. These clocks are statistical models that must first be trained and calibrated using individuals of known age and then can be used to estimate the biological or chronological age of new individuals from age-related methylation markers, most CpG sites but also potentially cytosines (e.g., CHG/CHH^102^) and, where relevant, other DNA modifications such as adenine methylation^103^.

Traditional age-estimation methods have limitations. Telomere length shows weak associations with age and is strongly influenced by environmental and physiological stressors^104^. Sclerochronological methods are widely used but is invasive. Phenotype, pigment and hormone markers resolve only broad age classification with low precision^105^. By contrast, DNA methylation-based epigenetic clocks provide higher accuracy and consistency^104^. The pan-tissue clock of Horvath demonstrated that methylation can predict ages across human tissues^58^. For non-human species, a systematic review by Tangili et al. identified 51 studies and reported 43 epigenetic clocks from 40 studies across diverse taxa, with a publication rate increase since 2018^106^. The clocks can also be built from multiple tissues (e.g. blood, skin, fin, muscle, liver, and gonads)^107,108^. Building on this, universal epigenetic clock refers to an age-prediction model that can estimate biological age across multiple tissues and species. Lu et al. developed a universal mammalian clock using 11754 methylation arrays spanning 59 tissues and 185 species^109^. They produced a chronological age clock, a relative age clock that normalizes age by species maximum lifespan, and a log–linear clock incorporating age at sexual maturity and gestation time. All models demonstrated high prediction accuracy, with a correlation coefficient (r) exceeding 0.95, supporting conserved aging-associated methylation signatures across mammals. For fish species, Polanowski et al. was the first study to establish an epigenetic clock for cetaceans by targeting specific loci using pyrosequencing^84^. Then the Bottlenose Dolphin Epigenetic Aging Tool (BEAT) utilizes DNA methylation levels in skin biopsy samples for age estimation in small cetaceans^85^. Similarly Mori et al. established a non-lethal method to estimate the chronological age of Risso’s dolphins (*Grampus griseus)* by analyzing DNA methylation in skin tissues^110^.

**Fig. 2:**
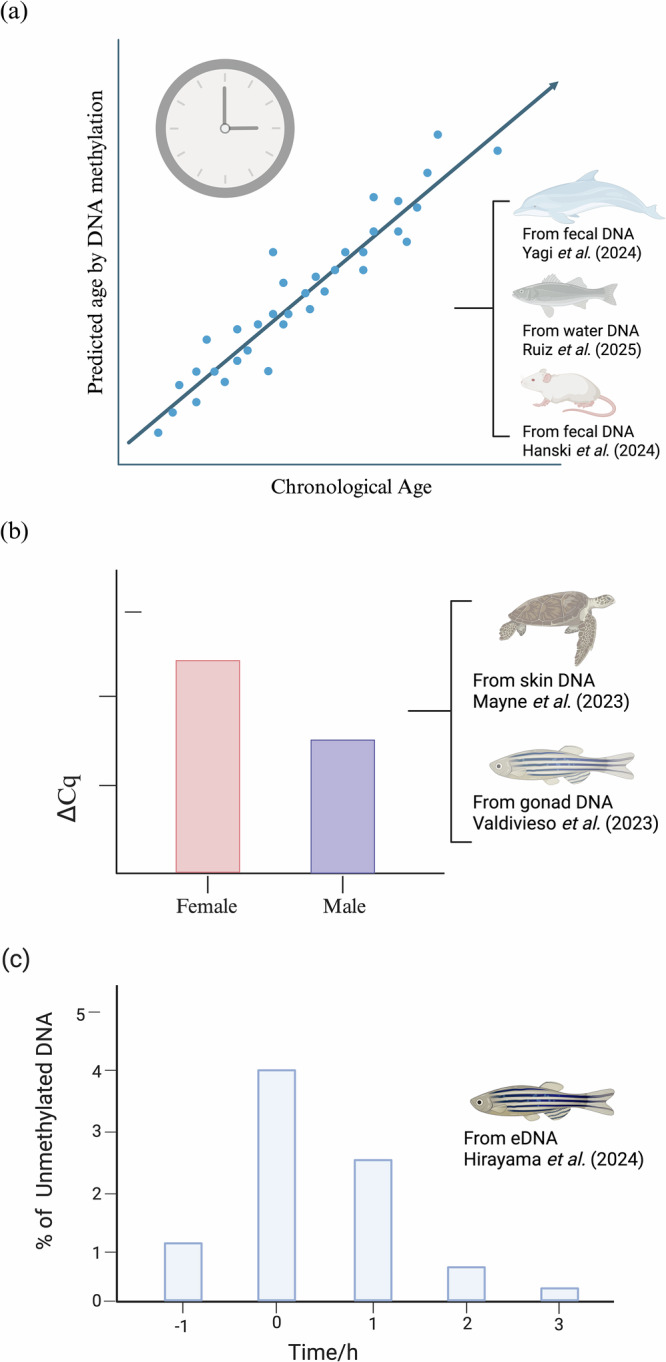
Applications of DNA methylation of target organisms. Some of the key applications of DNA methylation include inferring age and sex. **a** A diagram illustrating the relationship between chronological age and DNA methylation–based age. The diagram highlights the principle of epigenetic clocks, in which methylation signals derived from different biological sample types (e.g., skin, feces, or environmental DNA) are used to estimate biological age^92,111,112^. **b** A conceptual diagram illustrating sex-specific DNA methylation differences detected using targeted molecular assays (qPCR)^108,122^. Relative differences in methylation signals (ΔCq value) are shown schematically to indicate how males and females can be distinguished based on sex-biased methylation patterns. **c** A diagram showing the detection of unmethylated eDNA during a spawning event^100^. The proportion of unmethylated DNA increases due to the release of eggs from ovaries, which exhibit lower methylation levels. Figure 2 was created in BioRender. Chengbin, L. (2026) https://BioRender.com/lkxwe0b.

Estimating population age composition is one of the early applications of meth-eDNA. Yagi et al. demonstrated that methylation clocks from fecal samples of wild Indo-Pacific bottlenose dolphins (*Tursiops aduncus*) using MS-HRM analysis targeting *GRIA2*/*CDKN2A* achieved an accuracy of mean absolute error (MAE) of 5.08 years (10–13% of lifespan)^111^. Also, Hanski et al. established an epigenetic clock for house mice (*Mus musculus*) based on fecal DNA. After training on laboratory mice, the clock yielded highly accurate age predictions both in the training set (MAE = 23 days) and in the validation set (MAE = 26 days)^112^. Its application to wild mice revealed individual variation in epigenetic aging rates. Despite these differences, the clock successfully distinguished juveniles from adults in natural populations of house mice, marking one of the first demonstrations of fecal meth-eDNA for age inference in wild mammals. Other than the freshwater snail study^99^, Ruiz et al. developed an epigenetic clock using eDNA derived from aquaculture tanks of European seabass (*Dicentrarchus labrax*)^92^. Their model achieved a median age prediction error of only 2.6 days for larvae aged 10–24 days, an accuracy that is comparable to previously established tissue-DNA based epigenetic clocks in European seabass. Although nanopore sequencing enables the genome-wide detection of DNA methylation in the study, all age-associated differentially methylated sites were identified exclusively on the mitogenome. This mitochondrial restriction was attributed to coverage bias, as mitochondrial DNA is more abundant and better preserved in eDNA. While this constrains the direct application of the nuclear genome in tissue-based epigenetic clocks, it also creates a key opportunity for meth-eDNA, as species identification in eDNA surveys relies primarily on mitogenomes. Therefore, using mitochondrial DNA methylation may help both inference of species identification and age from environmental samples. The relationship between chronological age and predicted age by eDNA methylation rate have been shown as Fig. 2a.

### Sex identification and sex ratio monitoring

Sex-specific DNA methylation is a fundamental mechanism that underlies sexual differentiation across species. Fish exhibit sexual plasticity, with numerous species undergoing natural sex transitions during their lifetime or experiencing sex reversal in response to environmental factors, such as temperature, social cues, and stress^113^. Epigenetic mechanisms, particularly DNA methylation, play pivotal roles in both initiating and stabilizing these sex transitions (shown in Fig. 2b as a diagram)^108^. Given the high diversity and plasticity of sex in fish, methodologies that do not rely on genotyping are critical in eDNA analysis, where direct observation of individuals is unfeasible^114^.

Inhibition of DNA methylation prevents female-to-male transition in zebrafish as they lack heteromorphic sex chromosomes^115^. Likewise, studies in Pacific oysters (*Crassostrea gigas*), an invertebrate species lacking sex chromosomes, reveal that male gonads exhibit significantly higher methylation levels than female gonads^116^. Temperature-sensitive species like American alligator (*Alligator mississippiensis*) and tiger pufferfish (*Takifugu rubripes)*, whose sex is determined by incubation temperature, demonstrate that environmental cues can induce sex-specific methylation reprogramming, or facilitate sex reversal via methylation changes in key developmental genes^117,118^. Even in aphids (*Myzus persicae*), where males are haploid for the X chromosome, methylation patterns compensate for gene dosage, with X-linked genes hypermethylated and autosomal genes hypomethylated in males^119^. Altogether, these findings suggest that sex-associated DNA methylation is flexible and responsive to environmental and developmental contexts.

Although no published studies have yet applied meth-eDNA to population-level sex ratio inference, the concept is theoretically established. Many organisms exhibit sex-specific methylation patterns across the genome^117,120^. If DNA from sexually dimorphic loci is released and retained in environmental matrices, it should be possible to infer sex ratios based on the methylation status of eDNA. In Zhao et al., which detected the methylation rate of eDNA from freshwater snail, a hermaphroditic species was chosen to avoid confounding effects from sex-specific methylation^99^. It was noted that for gonochoristic species, sexually dimorphic methylation could be a target signal. Many reptiles, including turtles, exhibit temperature-dependent sex determination. In such species, genotyping alone cannot be used to reliably identify phenotypic sex^121^. Indeed, studies on green sea turtles have successfully utilized methylation profiles from skin tissue to determine sex (a diagram shown in Fig. 2b)^122^. These findings lay a methodological foundation for the future use of meth-eDNA in sex ratio estimation.

### Reproductive state and spawning detection

DNA methylation regulates gene expression during reproduction, affecting hormonal signaling, fertility, and spawning timing. Dynamic, stage-specific methylation patterns have been observed in gametogenesis across species, such as scallops (*Patinopecten yessoensis*) and oysters (*Crassostrea gigas*), where global methylation levels increase during the oogenesis and spermatogenesis stages, coinciding with high *DNMT3* expression^64,123^. For example, in zebrafish, promoter methylation of reproductive genes such as *esr1* (estrogen receptor alpha) and *amh* (anti-Müllerian hormone) is associated with sex-biased gene expression in both gonads and liver, while epigenetic regulators like *dnmt1*, *dnmt3*, and *hdac1* are more highly expressed in ovaries than in testes, indicating a sex-specific methylation landscape governing gonadal function^64,67^.

Sexual maturity prediction has been advanced using conserved CpG sites to develop predictive models for reproductive timing. By using tissue-derived DNA methylation signal. Heydenrych et al. demonstrated that CpG density in promoter regions can accurately predict sexual maturity age^124^. The correlation coefficients of their models were 0.81 in females and 0.76 in males, supporting the notion that methylation mediates the regulation of reproductive processes. This approach is particularly beneficial for endangered species or species that are difficult to observe over a long period of time in the wild.

Gestation and fertility have also been linked to DNA methylation through epigenetic clocks. Li et al. found that gestational duration correlated with methylation levels in placental tissues^125^, achieving a correlation coefficient = 0.96 in model predictions, emphasizing the potential of epigenetic clocks in reproductive ecology. Sperm quality and fertility are influenced by methylation as well: in striped bass, MBD-Seq identified 171 differential methylation regions (DMRs) distinguishing high- and low-fertility sperm^68^, while in common carp (*Cyprinus carpio*), in vitro sperm aging is associated with temporal methylation changes that correlate with declines in motility and fertilization rate, peaking at 24 hours post-stripping and declining by 96 hours^126^. The observed stage-specific methylation patterns during gametogenesis and regulation of hormonal pathways suggest they may contribute to coordinating reproductive cycles and energy allocation.

Meth-eDNA has also shown potential in reproductive ecology. Hirayama et al. demonstrated that spikes of unmethylated DNA^100^, a characteristic of germ cell genomes, appeared in water samples during the peak spawning periods (shown in Fig. 2c as a diagram). Since complementary unmethylated rDNA accounts for most rDNA repeats in unfertilized eggs, their release leads to a transient but detectable signature in eDNA, compared to the high methylation rate detected in somatic cells. Thus, the sharp shift in eDNA methylation signals can serve as an indicator of spawning events. While Hirayama et al. focused on transient spikes of unmethylated DNA associated with oocytes and eggs^100^, an analogous approach could, in principle, leverage sperm-specific hypermethylation as a complementary marker to infer spawning sites—if sperm (or sperm-derived DNA) is released in sufficient quantities and persists long enough to be detected. In practice, however, the detectability and direction of the methylation shift will depend on the relative contributions, degradation dynamics, and baseline methylation of somatic eDNA, so validation in target species and environments is required. Therefore, meth-eDNA is most directly applicable to detecting spawning events where germ-cell DNA is released into the environment, using either hypomethylated (egg-derived) or hypermethylated (sperm-derived) signatures.

### Health status and stress responses

Epigenetic modifications are responsive to physiological stress and disease, making meth-eDNA a potential indicator of ecosystem health, as it can reflect the current biological state of organisms. Balard et al. reviewed the potential of DNA methylation signals to assess health conditions in wildlife populations^37^. If specific methylation changes are linked to exposure to pollutants or pathogens, their detection in eDNA could act as an early warning signal of ecosystem stress or disease outbreaks. Although still in a conceptual phase, studies have shown that stressors such as chemical pollutants or infections can induce reproducible methylation changes^127^.

In marine mammals, anthropogenic stressors such as underwater noise and tourism pressure are of growing concern. While cortisol levels provide a measurement of short-term stress, DNA methylation analysis offers a non-invasive method for assessing medium- to long-term stress, which could be valuable for cetacean conservation^128^. For example, killer whales (*Orcinus orca*) were found to have distinct methylation patterns in stress-response genes between populations exposed to different levels of human activity^128^. In bottlenose dolphins (*Tursiops* spp.), DNA methylation-based epigenetic clocks not only estimated age but also showed that individuals with accelerated epigenetic aging—i.e., a higher DNA methylation age than expected—tended to have lower health scores that are significantly related to the survival probabilities^129^. These findings highlight the utility of DNA methylation as an indicator of age and health impacts, enabling more precise and long-term monitoring of cetacean populations under human pressure.

Meth-eDNA thus might offer a useful tool for monitoring organismal response to environmental stress, particularly in scenarios where direct biological sampling in the field is not feasible. For example, changes of temperature and salinity can induce methylation changes in promoter regions in crustaceans^130^ and fish^131^. Promoters with varying CpG densities are associated with distinct gene functions, and alterations in their methylation states—detected through meth-eDNA—can be used to infer whether organisms are experiencing stress and to assess stress level. However, there are differences in methylation patterns and functions among different species^132^, and the broad applicability of meth-eDNA still needs further verification. In line with this concept, Hishikawa et al. reported that accelerated DNA methylation age and increased DNA damage in urinary shedding cells are significantly correlated with current renal function. Studies on patients with chronic kidney disease showed that epigenetic age acceleration—measured using Hannum’s and PhenoAge clocks—was strongly associated with both reduction of estimated glomerular filtration rate and its rate of decline^133^. These clinical findings, though focused on human kidney health, highlight the potential of methylation-based signatures in excreted DNA to monitor stress noninvasively. As sensitivity improves and new methylation signals are validated, it may become feasible to monitor population health and environmental stressors through meth-eDNA analysis.

### Conclusions and perspectives

In summary, research on DNA methylation in environmental DNA (meth-eDNA) has just emerged in recent years. From its conceptual inception in 2019, when researchers first proposed that methylated cytosines in eDNA could be detected in environmental samples^98^, several empirical studies in recent years support the idea^92,99,100,111,112^. Recent studies confirm that meth-eDNA may stably capture functional information at both the individual and population levels. Although these studies have been conducted in aquarium tanks or using specific environmental samples, these findings position meth-eDNA as a promising method for ecological inference. To facilitate translation of these advances, we summarize key priorities and next steps for meth-eDNA research in Box 1. Looking forward, continued advances in sequencing technologies and methylation detection methods will unlock more potential of meth-eDNA in ecological monitoring, resource management, and biodiversity conservation.

Box 1. Key priorities for advancing Meth-eDNA research **Conceptual shift**

- Meth-eDNA extends eDNA to functional ecological inference, thus giving population-level insights into age structure, sex ratios, reproduction, and health status.
**Methodological requirements**
- Standardized workflows for methylation detection in degraded eDNA
- Validation frameworks comparing tissue DNA and matched eDNA methylation profiles
- Identification of specific methylation markers

**Ecological application**

- Age structure inference using epigenetic clocks
- Sex ratio monitoring based on sex-specific methylation patterns
- Spawning detection through germ-related methylation markers
- Health and stress monitoring using environmentally responsive methylation markers

**Current challenges**

- Low-quantity and quality of DNA in environmental samples because of DNA degradation and dispersion in the environment
- Limited reference methylomes across species
- Uncertainty in interpreting population-level signals from mixed eDNA

**Future direction**

- Broadening species methylation reference datasets
- Development of methylation sequencing approaches for ecosystem monitoring
- Understanding of the dynamics of meth-eDNA such as the stability of methylation signal under field conditions
- Expansion from controlled tank experiments to field ecological validation

### Reporting summary

Further information on research design is available in the Nature Portfolio Reporting Summary linked to this article.

## Supplementary information

Reporting Summary
